# Regulation of Erythropoietin Receptor Activity in Endothelial Cells by Different Erythropoietin (EPO) Derivatives: An *in Vitro* Study

**DOI:** 10.3390/ijms14022258

**Published:** 2013-01-24

**Authors:** Maria Letizia Trincavelli, Eleonora Da Pozzo, Osele Ciampi, Serena Cuboni, Simona Daniele, Maria Pia Abbracchio, Claudia Martini

**Affiliations:** 1 Department of Pharmacy, University of Pisa, Pisa 56126, Italy; E-Mails: dapozzo@farm.unipi.it (E.D.P.); osele.ciampi@marionegri.it (O.C.); serena_cuboni@yahoo.it (S.C.); simona.daniele@for.unipi.it (S.D.); 2 Department of Pharmacological Sciences, University of Milan, Milan 20133, Italy; E-Mail: mariapia.abbracchio@unimi.it

**Keywords:** endothelial cells, erythropoietin receptor, erythropoiesis-stimulating agents, STAT-5 pathway, receptor desensitization, signal resensitization, cell proliferation, angiogenesis

## Abstract

In endothelial cells, erythropoietin receptors (EPORs) mediate the protective, proliferative and angiogenic effects of EPO and its analogues, which act as EPOR agonists. Because hormonal receptors undergo functional changes upon chronic exposure to agonists and because erythropoiesis-stimulating agents (ESAs) are used for the long-term treatment of anemia, it is critical to determine the mechanism by which EPOR responsiveness is regulated at the vascular level after prolonged exposure to ESAs. Here, we investigated EPOR desensitization/resensitization in human umbilical vein endothelial cells (HUVECs) upon exposure to three ESAs with different pharmacokinetic profiles, epoetin alpha (EPOα), darbepoetin alpha (DarbEPO) and continuous EPOR activator (CERA). These agonists all induced activation of the transcription factor STAT-5, which is a component of the intracellular pathway associated with EPORs. STAT-5 activation occurred with either monophasic or biphasic kinetics for EPOα/DarbEPO and CERA, respectively. ESAs, likely through activation of the STAT-5 pathway, induced endothelial cell proliferation and stimulated angiogenesis *in vitro*, demonstrating a functional role for epoetins on endothelial cells. All epoetins induced EPOR desensitization with more rapid kinetics for CERA compared to EPOα and DarbEPO. However, the recovery of receptor responsiveness was strictly dependent on the type of epoetin, the agonist concentration and the time of exposure to the agonist. EPOR resensitization occurred with more rapid kinetics after exposure to low epoetin concentrations for a short period of desensitization. When the highest concentration of agonists was tested, the recovery of receptor responsiveness was more rapid with CERA compared to EPOα and was completely absent with DarbEPO. Our results demonstrate that these three ESAs regulate EPOR resensitization by very different mechanisms and that both the type of molecule and the length of EPOR stimulation are factors that are critical for the control of EPOR functioning in endothelial cells. The differences observed in receptor resensitization after stimulation with the structurally different ESAs are most likely due different control mechanisms of receptor turnover at the intracellular level.

## 1. Introduction

Erythropoietin (EPO) is a glycoprotein hormone that is the primary regulator of erythropoiesis [1,2]. EPO acts via interaction with a single chain cell surface receptor of the cytokine receptor superfamily [3]. The EPO receptor (EPOR) is expressed at high levels on erythroid progenitor cells and is the primary target for EPO binding [4]. However, EPORs have also been detected and demonstrated to be functionally active on non-hematopoietic cells, including endothelial cells and endothelial progenitor cells (EPCs) [5–8]. Therefore, the endothelium is considered a physiological target of EPO [9]. EPO is involved in the recruitment and mobilization of endothelial progenitors that orchestrate vascular reparative processes and promote endothelial regeneration. In addition, EPO has the following important direct effects on mature endothelial cells: (i) stabilizes endothelial structures and vascular integrity, such as cell-cell and cell-matrix contacts; (ii) has an important protective action under hypoxic conditions by increasing endothelial cell proliferation and protecting cells against ischemia and apoptosis caused by inflammatory cytokines [10–14] and (iii) stimulates angiogenesis in *in vitro* and *in vivo* models, thereby acting as a bona fide direct angiogenic factor [15–20]. These data have laid the groundwork for exploring the therapeutic potential of recombinant human EPO and its analogous in vascular medicine and their consolidated use in the treatment of anemic states.

The tissue-protective effects of EPO are mediated by ligand-induced receptor activation and the recruitment of specific intracellular signaling pathways, including the JAK2/STAT-5 and PI3K/Akt protein kinases [21–24]. The first signaling molecule that binds to ligand-activated receptors and induces an intracellular signaling cascade is the JAK2 tyrosine kinase, a cytoplasmic tyrosine kinase that phosphorylates tyrosine residues within the EPOR itself. EPOR phosphorylation provides docking sites for a variety of signaling molecules, such as STAT-5, PI3K and MAPK. These activated kinases translocate to the nucleus and induce the transcription of target genes that are involved in both the inhibition of apoptosis and cell proliferation. STAT-5 has also been described as the major transcription factor that is involved in EPO-mediated proliferative and angiogenic effects on endothelial cells [15,24,25]. In addition, the beta-common receptor, which is a shared receptor subunit for the interleukin-3, interleukin-5 and GM-CSF receptors, has been demonstrated to mediate several non-hematopoietic effects of EPO in endothelial cells, such as eNOS activation and angiogenesis [26].

All known erythropoietin-stimulating agents act as EPOR agonists [27,28], and their clinical efficacy is highly dependent on the functional responsiveness of surface membrane receptors, which are known to be modulated in both physiological and pathological conditions by specific and complex regulatory mechanisms.

Some of these mechanisms regulate receptor abundance and/or availability, whereas others may alter the responsiveness of downstream signaling molecules to receptor engagement. These alterations in receptor responsiveness include receptor desensitization, downregulation and resensitization. These mechanisms are critical for the regulation of temporal and spatial aspects of receptor signaling and serve to ensure that extracellular stimuli are translated into intracellular signals of the appropriate magnitude, duration and specificity.

Negative regulatory pathways that lead to the termination of intracellular signals are simultaneously turned on by EPO itself. These negative signaling events include receptor de-phosphorylation by the phosphotyrosine-phosphatase SHP1 [29], internalization and degradation of the activated receptor [30–34] and receptor downregulation induced by lysosomes and the proteasome [35–37]. Therefore, studying receptor activation following treatment with drugs represents an important approach to identify novel therapeutic strategies and clarify the differences between structurally related drugs at the molecular level.

In this study, we investigated the effects of different epoetin derivatives, epoetin alpha (EPOα), darbepoetin (DarbEPO) and continuous EPOR activator (CERA), on EPOR desensitization and signaling resensitization. We also specifically evaluated EPOR-mediated activation of intracellular STAT-5 in HUVECs. In addition, we determined the effects of EPO derivatives on HUVEC angiogenesis and viability and involvement of the STAT-5 pathway in these functional effects. These results could elucidate the different signaling pathways that are activated by EPO derivatives at the molecular level and may also provide information regarding the therapeutic potential of these drugs in endothelial repair and regeneration.

## 2. Results

### 2.1. Optimization of Experimental Conditions

**Figure 1:**
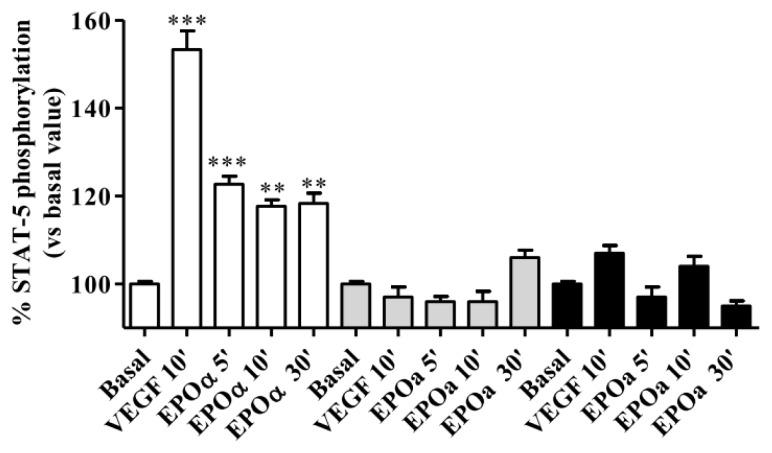
Vascular endothelial growth factor (VEGF)- and epoetin alpha (EPOα)-mediated STAT-5 phosphorylation. Human umbilical vein endothelial cells (HUVECs) were treated with 25 ng/mL VEGF (10 min) or 1 IU/mL EPOα (5–10 and 30 min) under three different cell culture conditions. White bar: replacement of cell culture medium for 24 h with M199 without fetal bovine serum (FBS) and growth factors supplemented with 0.5% bovine serum albumin (BSA) and 0.2 mM orthovanadate. Gray bars: replacement of cell culture medium for 24 h with M199 without FBS and growth factors. Black bars: replacement of cell culture medium for 12 h with M199 medium without FBS and growth factors supplemented with 0.2 mM orthovanadate 30 min before the experiments. In each experimental condition, cell responsiveness was evaluated by assessing whether VEGF or EPOα stimulated STAT-5 phosphorylation. The data are expressed as the percent STAT-5 phosphorylation compared to the untreated control cells (set to 100%) and represent the mean ± SEM of three different experiments. *** *p* < 0.001; ** *p* < 0.01 *vs.* basal value.

EPOα-induced STAT-5 phosphorylation (1 IU/mL for 5, 10 and 30 min) was evaluated by maintaining HUVECs under three different culture conditions, as reported in the Materials and Methods section, and 25 ng/mL VEGF was used as a positive control. The results showed that 1 IU/mL EPOα stimulated STAT-5 phosphorylation in a time-dependent manner in HUVECs maintained in M199 medium devoid of FBS and growth factors and supplemented with 0.5% BSA and 0.2 mM OVD for 24 h before the experiment (Figure 1). These results suggest that the addition of BSA, which has trophic effects, and OVD, which reduces protein de-phosphorylation, produces the ideal experimental conditions to assess EPOR responsiveness to EPOα. The same results were obtained by treating cells with VEGF. Based on these results, these experimental conditions were used for all subsequent experiments.

### 2.2. EPOα-, DarbEPO- and CERA-Mediated STAT-5 Phosphorylation: Concentration- and Time-Dependence

**Figure 2:**
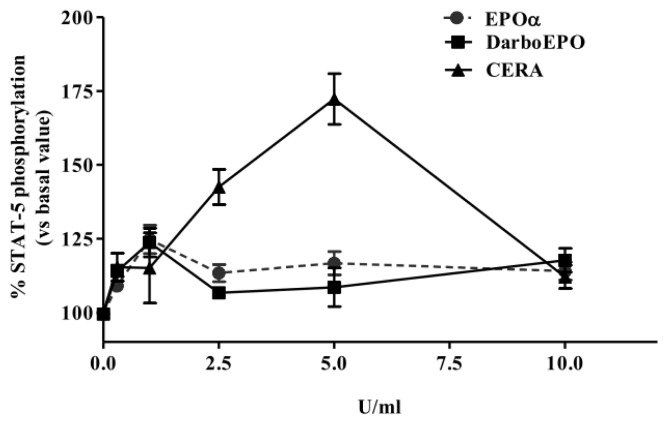
Concentration-dependence of EPOα-, darbepoetin alpha (DarbEPO)- and continuous erythropoietin receptors activator (CERA)-mediated STAT-5 phosphorylation. HUVECs were treated with different epoetin concentrations (0.5–10 IU/mL) for 5 min. STAT-5 phosphorylation levels were quantified using an enzyme-linked immunosorbent assay (ELISA) kit (see Materials and Methods). The data are expressed as the percent STAT-5 phosphorylation compared to the untreated control cells (set to 100%) and represent the mean ± SEM of three different experiments.

HUVECs were pre-incubated for 24 h in M199 medium supplemented with 0.5% BSA and 0.2 mM OVD. The cells were then treated for five minutes with various concentrations of EPOα, DarbEPO or CERA (0.5 to 10 IU/mL). A control sample was used for each experimental condition to obtain the basal value (Figure 2). The results demonstrate that all tested epoetins stimulated STAT-5 phosphorylation in a concentration-dependent manner with a maximal effect at 1 IU/mL for EPOα and DarbEPO and 5 IU/mL for CERA. No significant differences between the EPOα and DarbEPO dose-response curves were detected, whereas at the highest concentration of CERA, higher maximal STAT-5 phosphorylation was induced (Figure 2).

**Figure 3:**
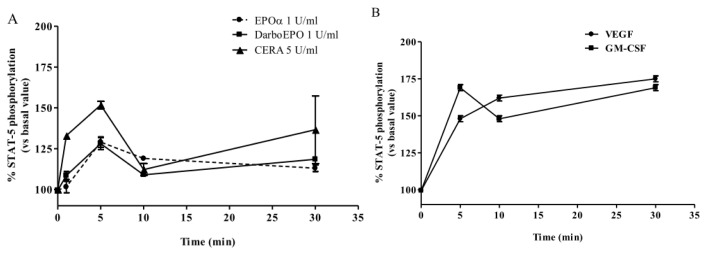
Time-dependence of EPOα-, DarbEPO- and CERA-mediated STAT-5 phosphorylation. HUVECs were treated with medium alone (control) or 1 IU/mL EPOα, 1 IU/mL DarbEPO or 5 IU/mL CERA for different periods of time ranging from 5 to 30 min (**A**). Aliquots of cells were treated with VEGF (25 ng/mL) or granulocyte-macrophage colony-stimulating factor (GM-CSF) (10 IU/mL) for the same time intervals as above (**B**). STAT-5 phosphorylation levels were quantified using an ELISA kit (see Materials and Methods). The data are expressed as the percent STAT-5 phosphorylation compared to the untreated control cells (set to 100%) and represent the mean ± SEM of three different experiments.

The time-course of EPOα- and DarbEPO**-**mediated STAT-5 phosphorylation was also evaluated. After 24 h in the appropriate culture medium, HUVECs were treated with the epoetins at the concentration causing the maximal effect (1 IU/mL for EPOα and DarbEPO; 5 IU/mL for CERA) for 1, 5, 10 and 30 min. A control sample was used for each experimental condition to obtain the basal value (Figure 3A). The results demonstrate that 1 IU/mL EPOα and 1 IU/mL DarbEPO stimulated STAT-5 phosphorylation in a similar, time-dependent manner with rapid and transient kinetics and a maximal effect after treatment for 5 min. Cell treatment with CERA induced a biphasic activation of STAT-5 with two peaks at 5 and 30 min (Figure 3A). To investigate STAT-5 activation kinetics in detail, similar experiments were performed using 25 ng/mL VEGF and 10 IU/mL GM-CSF as positive controls (Figure 3B). The data confirmed that VEGF induces time-dependent activation of STAT-5 with a maximal effect after 30 min. GM-CSF showed a biphasic effect similar to CERA. These results suggest that the kinetics of STAT-5 activation follow different time-courses depending on the type of epoetin used to induce activation.

**Figure 4:**
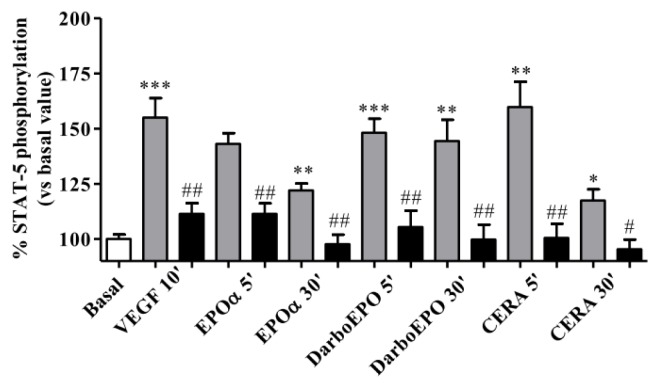
Effect of the STAT-5 inhibitor on EPOα-, DarbEPO- and CERA-mediated STAT-5 phosphorylation. HUVECs were treated with medium alone (basal) or 1 IU/mL EPOα, 1 IU/mL DarbEPO or 5 IU/mL CERA for 5–30 min or VEGF for 10 min in the absence (gray bar) or presence of the STAT-5 inhibitor (80 μM, black bar). STAT-5 phosphorylation levels were quantified using an ELISA kit (see Materials and Methods). The data are expressed as the per cent STAT-5 phosphorylation compared to the untreated control cells (set to 100%) and represent the mean ± SEM of three different experiments. *** *p* < 0.001; ** *p* < 0.01: * *p* < 0.05 *vs*. basal value. ### *p* < 0.001; ## *p* < 0.01 *vs.* control (in the absence of inhibitor).

To verify the specificity of the results, the same experiments evaluating the effects of VEGF and the different ESAs on STAT-5 phosphorylation were performed in the presence of a STAT-5 inhibitor *N′*-((4-Oxo-4H-chromen-3-yl)methylene)nicotinohydrazide. This compound is a cell permeable non-peptidic nicotinoyl hydrazone that selectively targets the SH2 domain of STAT-5 (IC_50_ = 47 μM) and has a much less potent effect towards the SH2 domain of STAT-1, STAT-3 or Lck (IC_50_ > 500 μM) [38]. The results depicted in Figure 4 show that the STAT-5 inhibitor completely prevented VEGF- and ESA-mediated activation of STAT-5 phosphorylation, demonstrating that VEGF- and ESA-mediated activation of STAT-5 phosphorylation is a specific effect.

### 2.3. Effect of EPOα, DarbEPO and CERA on HUVEC Viability

**Figure 5:**
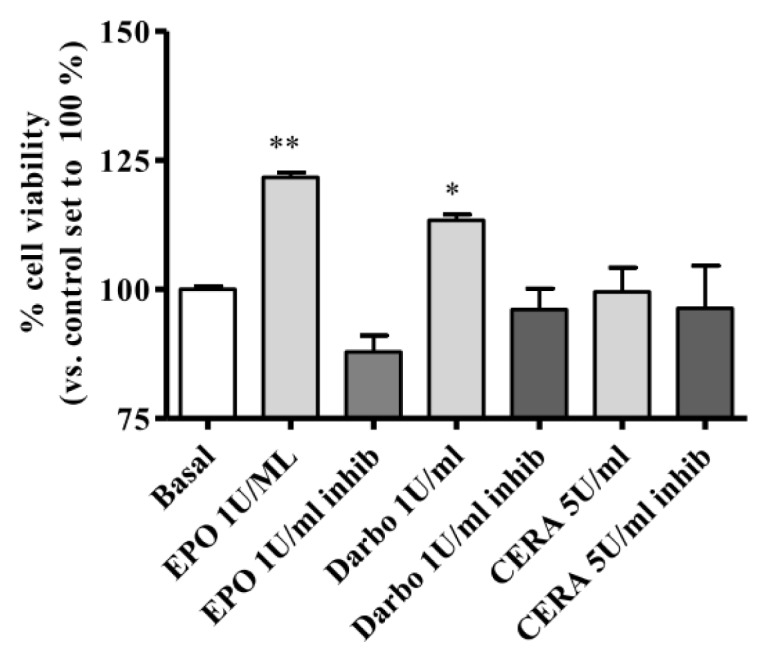
Effect of erythropoiesis-stimulating agents (ESAs) on HUVEC viability. The cells were treated for 72 h with EPOα (1 IU/mL), DarbEPO (1 IU/mL) or CERA (5 IU/mL) in the absence or presence of 80 μM STAT-5 inhibitor. Cell viability was determined by an MTS assay, as described in the Methods section. The data are expressed as the percent cell viability compared to the untreated basal cells (set to 100%) and represent the mean ± SEM of three different experiments. ** *p* < 0.01; * *p* < 0.05 *vs.* basal value.

To verify the role of the different ESAs in endothelial cell viability, an MTS assay was used to analyze metabolically active cells after treatment with the different ESA derivatives in the absence or presence of the STAT-5 inhibitor. As shown in Figure 5, EPOα and DarbEPO induced a significant increase in cell viability compared to the untreated control cells (EPOα: 121.7% ± 0.9%, *p* < 0.01; DarbEPO: 113.4% ± 1.1%, *p* < 0.05 *vs.* basal value set to 100%). These effects appeared to be completely prevented following incubation of the cells with the STAT-5 inhibitor, suggesting that the STAT-5 pathway is primarily involved in the EPOα- and DarbEPO-mediated effects on cell viability. By contrast, 5 IU/mL CERA treatment for 72 h had no significant effect on cell viability.

### 2.4. EPOα-, DarbEPO- and CERA-Mediated Angiogenesis of HUVECs *In Vitro*

**Figure 6:**
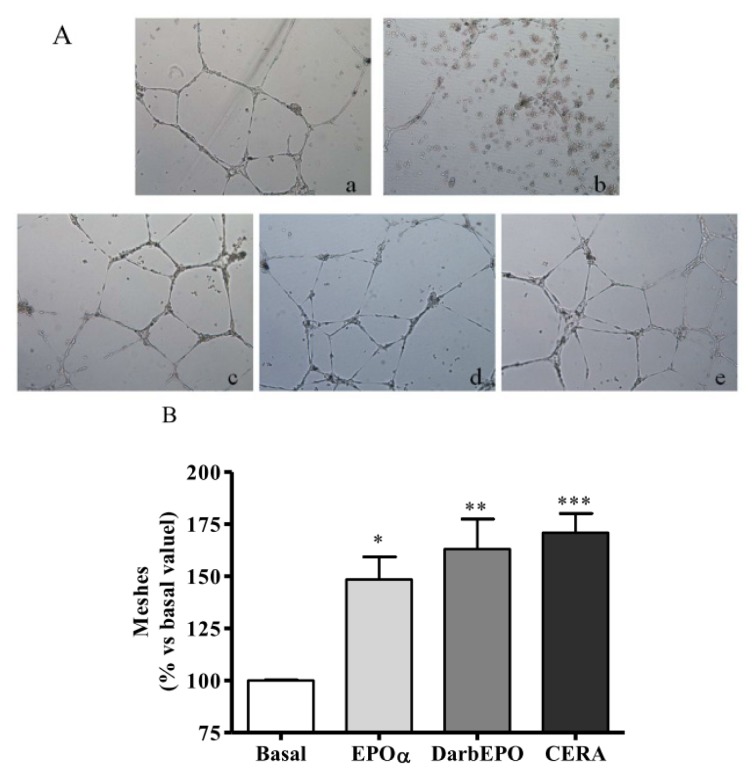
Effect of ESAs on HUVEC angiogenesis. The cells were treated with medium alone (basal) or ESAs **(**1 IU/mL EPOα, 1 IU/mL DarbEPO or 5 IU/mL CERA) for 24 h before seeding onto Matrigel with fresh medium. Capillary-like tube formation was observed by microscopy and quantified using the ImageJ program. (**A**) Representative pictures of HUVEC tubule formation on Matrigel after 24 h drug incubation. (**a**) Basal; (**b**) STAT-5 inhibitor; (**c**) 1 IU/mL EPOα; (**d**) 1 IU/mL DarbEPO; (**e**) 5 IU/mL CERA (original magnification = 10×). (**B**) The number of mesh-like structures were quantified and expressed as a percent of the control sample. The data are expressed as the mean ± SEM of three independent experiments performed in duplicate. *** *p* < 0.001; ** *p* < 0.01; * *p* < 0.05 *vs.* basal value.

To determine whether ESA derivatives could affect the angiogenic property of endothelial cells, we performed a capillary-like tube formation assay on Matrigel. This is a commonly used method for the evaluation of i*n vitro* angiogenesis. HUVECs were pre-treated for 24 h with 1 IU/mL EPOα, 1 IU/mL DarbEPO or 5 IU/mL CERA in the absence or presence of 80 μM STAT-5 inhibitor. The cells were then seeded onto Matrigel in 24-well plates with fresh medium. The capillary-like tube formation assay was developed, and photos were taken after a 20 h incubation (Figure 6A). Quantitative analysis revealed that the number of mesh-like structures following treatment with the three ESAs was significantly higher compared to the untreated control (Figure 6B, EPOα 148.4 ± 26.8, *p* < 0.05; DarbEPO 163.1 ± 35.3, *p* < 0.01; CERA 170.8 ± 26.3, *p* < 0.001 *vs.* basal value). These data suggest that EPOα, DarbEPO and CERA promoted the angiogenesis of HUVECs *in vitro*. However, the STAT-5 inhibitor alone induced a significant inhibitory effect on capillary tube formation (data not shown) and was, therefore, not suitable under our experimental conditions to verify the involvement of STAT-5 in ESA-mediated pro-angiogenic activity.

### 2.5. EPOR Desensitization

**Figure 7:**
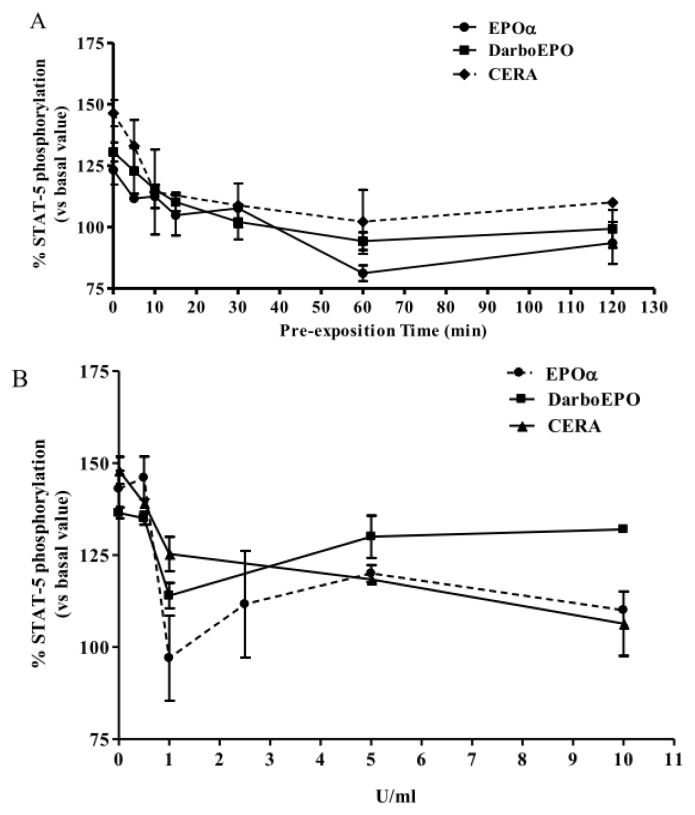
EPOR desensitization. (**A**) Time-dependence: cells were pre-treated with medium alone (control) or 1 IU/mL EPOα, 1 IU/mL DarbEPO or 5 IU/mL CERA for different periods of time ranging from 5 to 120 min. (**B**) Concentration-dependence: cells were pre-treated with medium alone (control) or different EPOα, DarbEPO or CERA concentrations (0.5–10 IU/mL) for 60 min. The cells were then washed and stimulated by the addition of 1 IU/mL EPOα, 1 IU/mL DarbEPO or 5 IU/mL CERA for 5 min. STAT-5 phosphorylation levels were quantified using an ELISA kit (see Materials and Methods). The data are expressed as the percent STAT-5 phosphorylation compared to the untreated control cells (set to 100%) and represent the mean ± SEM of three different experiments.

The susceptibility of EPOR to desensitization upon prolonged agonist exposure and the time-dependence of this phenomenon were evaluated by incubating HUVECs with 1 IU/mL EPOα, 1 IU/mL DarbEPO or 5 IU/mL CERA for graded time intervals ranging from 5 to 120 min (Figure 7A). The results demonstrate that EPOα (1 IU/mL) treatment induced time-dependent receptor desensitization with a maximal effect after 60 min and a *t*_1/2_ value of 18.17 min. Similarly, DarbEPO (1 IU/mL) treatment desensitized EPORs with a *t*_1/2_ value of 11.21 min. Statistical analysis showed no significant differences in the desensitization kinetics of these two epoetins. By contrast, CERA induced EPOR desensitization with more rapid kinetics compared to the other epoetins (*t*_1/2_ = 2.77 min).

The data from the different experiments were normalized by concurrently performing an immunoenzymatic assay to quantify the total amount of STAT-5 protein. The results indicate that the various cell treatments did not alter the total protein levels of STAT-5 (data not shown).

In a similar set of experiments, the concentration-dependence of EPOR desensitization was assessed by exposing the HUVECs to different concentrations of EPOα, DarbEPO or CERA (0.5 to 10 IU/mL) for a fixed period of time (60 min) (Figure 7B). The results demonstrate that the three epoetins induced EPO receptor desensitization in a concentration-dependent manner. EPOα and DarbEPO desensitization appeared maximal at 1 IU/mL and tended to decrease at higher ligand concentrations, whereas CERA induced a concentration-dependent effect at all tested concentrations. These results suggest that CERA regulates EPOR signaling differently than EPOα and DarbEPO.

### 2.6. EPOR Resensitization

The resensitization of desensitized EPOR was then investigated following the induction of receptor desensitization with EPOα, DarbEPO or CERA at different concentrations for different exposure times (18 or 54 min). In the first set of experiments, HUVECs were pre-incubated with 1 IU/mL EPOα or DarbEPO for 18 or 54 min to induce receptor desensitization. In the second set of experiments, HUVECs were desensitized for the same period of time by pre-treating the cells with 3 IU/mL EPOα, 3 IU/mL DarbEPO or 5 IU/mL CERA. Following desensitization, the cells were washed to remove the ligands and placed in agonist-free medium for various times to allow for receptor resensitization (wash-out). EPOR responsiveness in the control untreated cells, in the desensitized cells and following resensitization was then evaluated by assessing EPOα- (1 IU/mL for 5 min) DarbEPO- (3 IU/mL for 5 min) or CERA-mediated (5 IU/mL for 5 min) phosphorylation of STAT-5.

**Figure 8:**
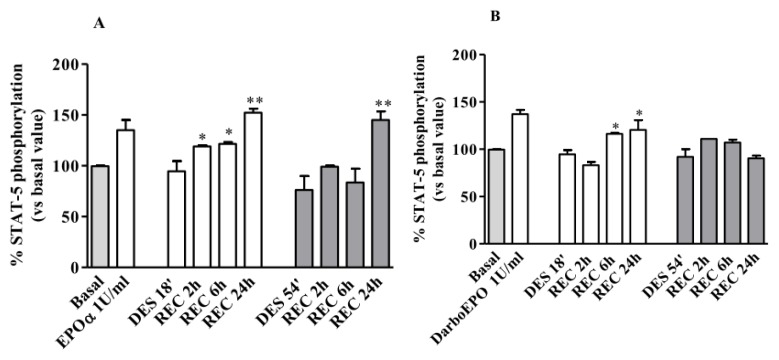
EPOR resensitization following 1 IU/mL EPOα- or DarbEPO-induced receptor desensitization. HUVECs were pre-treated with medium alone (control) or 1 IU/mL EPOα (**A**) or 1 IU/mL DarbEPO (**B**) for 18 or 54 min to induce receptor desensitization. Then, cells were the washed and replaced in medium alone for different periods of time (2 to 24 h) to allow for receptor resensitization. All samples were then stimulated by the addition of 1 IU/mL EPOα or 1 IU/mL DarbEPO for 5 min and STAT-5 phosphorylation levels were quantified. The data are expressed as the percent STAT-5 phosphorylation compared to the untreated control cells (set to 100%) and represent the mean ± SEM of three different experiments. ** *p* < 0.01; * *p* < 0.05 *vs.* DES’.

As expected, following treatment with 1 IU/mL EPO, receptor desensitization occurred in a time-dependent manner (Figure 8A). Recovery of receptor responsiveness was evaluated following cell wash-out for 2–24 h. As depicted in Figure 5A, when EPOR was desensitized for 18 min with EPOα (1 IU/mL), receptor resensitization began after a 2 h wash-out period and was complete after 24 h. When receptor desensitization was induced for 54 min with EPOα (1 IU/mL), receptor resensitization was detected only after a 24 h wash-out period. As expected, these results suggest that the kinetics of receptor resensitization depend on the duration of receptor exposure to the agonist and is slower when receptor desensitization is induced by a longer exposure to the agonist.

Concurrently, we evaluated EPOR resensitization following induction of desensitization with DarbEPO at the same concentrations. As shown in Figure 8B, following treatment of the HUVECs with DarbEPO (1 IU/ml) for 18 min, receptor responsiveness was restored only after a 6 hour wash-out period. Moreover, when the cells were treated with DarbEPO for 54 min, receptor responsiveness remained absent after a 24 h wash-out period, demonstrating that for this DarbEPO exposure time, receptor resensitization does not occur within the time period evaluated in this study. It is also evident that EPOR resensitization kinetics depend on the type of agonist used in the pre-exposure phase. During the desensitization period, receptor responsiveness recovery was more rapid with EPOα compared to DarbEPO.

**Figure 9:**
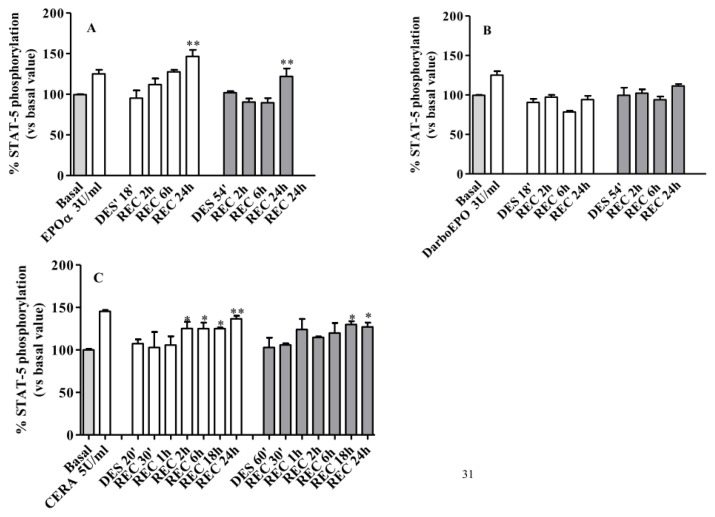
EPOR resensitization following 3 IU/mL EPOα-, 3 IU/mL DarbEPO- or 5 IU/mL CERA-induced receptor desensitization. HUVECs were pre-treated with medium alone (control) or 3 IU/mL EPOα (**A**), 3 IU/mL DarbEPO (**B**) or 5 IU/mL CERA (**C**) for 18 or 54 min to induce receptor desensitization. The cells were then washed and replaced in medium alone for different periods of time (2 to 24 h) to allow for receptor resensitization. All samples were then stimulated by the addition of 3 IU/mL EPOα, 3 IU/mL DarbEPO or 5 IU/mL CERA for 5 min, and STAT-5 phosphorylation levels were quantified. The data are expressed as the percent STAT-5 phosphorylation compared to the untreated control cells (set to 100%) and represent the mean ± SEM of three different experiments. ** *p* < 0.01; * *p* < 0.05 *vs.* DES’.

These experiments were also performed using higher agonist concentrations. As depicted in Figure 9A, when receptor desensitization was induced with the higher concentration of EPOα (3 IU/mL), the resensitization kinetics were slower with significant recovery only after a 24 h wash-out period. Under these conditions, no significant differences were observed following either the 18 or 54 min desensitization. When receptor desensitization was induced with DarbEPO (3 IU/mL), the receptor remained desensitized after the 24 h cell wash-out period without any recovery of responsiveness (Figure 9B).

The same experiments were performed following induction of EPOR desensitization with 5 IU/mL CERA. As shown in Figure 9C, following treatment with CERA (5 IU/mL) for 18 min, receptor responsiveness was restored after a 2 h wash-out period. By contrast, when the HUVECs were treated with CERA for 54 min, receptor responsiveness was recovered only after a 18–24 h wash-out period. This finding demonstrates that with CERA, the resensitization kinetics also depend on the duration of agonist exposure. By comparing the kinetics of EPOR resensitization obtained with the three epoetins, it is evident that CERA, when used at higher concentrations, allows for faster recovery of receptor responsiveness compared to EPOα and DarbEPO.

Overall, these results demonstrate that EPOR resensitization kinetics depend on the type of epoietin, its concentration and the length of time for desensitization. For both EPOα and DarbEPO, the resensitization kinetics are faster at lower concentrations and shorter exposure times. For CERA, the resensitization kinetics are quite rapid at high agonist concentrations. Our data also suggest that the regulation of EPOR desensitization/recycling by these three different epoetins is significantly different at the molecular level.

### 2.7. Effects of ESAs on EPOR Expression Levels

To evaluate whether there are any changes in EPOR at the mRNA and protein levels during EPOR desensitization/resensitization, real-time PCR and immunoblotting studies were performed.

**Figure 10:**
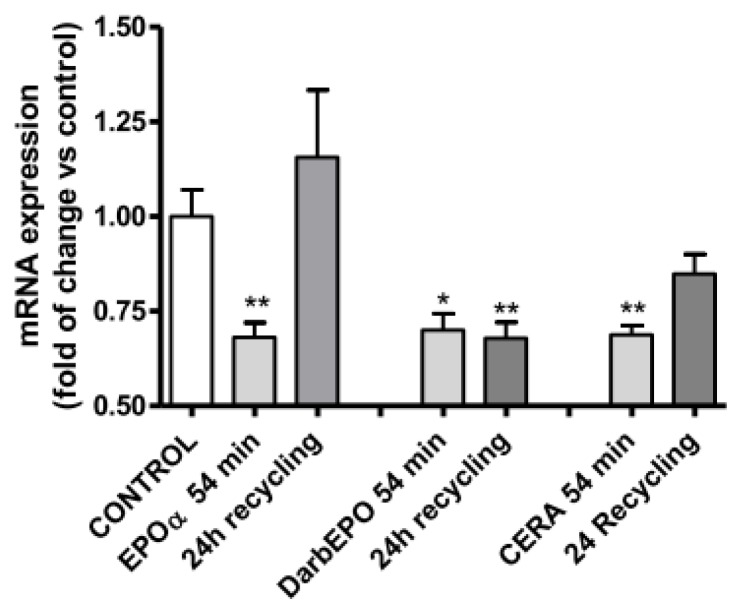
Effect of EPOR desensitization/resensitization on EPOR mRNA expression levels. The cells were pre-treated with medium alone (control) or 1 IU/mL EPOα, 1 IU/mL DarbEPO or 5 IU/mL CERA for 54 min and then washed-out for 24 h. RT-PCR for the EPOR gene was performed, and β-actin used as the house-keeping gene. The data are expressed as the fold mRNA EPOR changes *vs.* control. *p < 0.05, **p < 0.01 *vs*. control cells.

Using RT-PCR, we demonstrated that treatment of HUVECs with EPOα, DarbEPO or CERA for 54 min, which allowed for complete receptor desensitization, induced a significant reduction in EPOR mRNA expression without any significant difference among the three EPO derivatives. When the cells were resensitized in the absence of agonists for 24 h, a marked difference in epoetin-mediated regulation of EPOR mRNA was observed. When the cells were desensitized with EPOα or CERA and then subjected to resensitization, EPOR mRNA levels returned to basal levels. By contrast, after receptor desensitization with DarbEPO and subsequent cell resensitization, EPOR mRNA levels remained low (Figure 10). These data suggest that epoetins control transcription of EPOR by different mechanisms at the nuclear level. Furthermore, these differences may correlate with the differences observed in EPOR recycling on the plasma membrane after receptor activation by the three EPO derivatives.

## 3. Discussion

Until recently, EPO has primarily been regarded as the hematopoietic cytokine required for the survival, proliferation and differentiation of committed erythroid progenitor cells [1,4]. Treatment with EPO and its analogous has, therefore, represented a major breakthrough in the therapy of anemic states. However, EPO shows pleiotropic and regenerative properties in various other tissues, including the endothelium and is also becoming relevant to cardiovascular medicine [9].

Much conflicting information has been reported regarding the benefit/risk ratio resulting from the action of EPO on endothelial cells. Several groups have demonstrated that long-term therapy with EPO may induce increases in markers of endothelial injury and may favor platelet aggregation in hemodialyzed patients by shifting the balance of the haemostatic system towards thrombosis [39–42]. Theoretically, an increased hematocrit level could contribute to elevated blood pressure and/or vascular thrombosis. By contrast, recent data have demonstrated that EPO therapy does not affect coagulation activation [43]. High levels of EPO can reduce ischemia-reperfusion injury in the heart through an immediate response involving EPO-stimulation of nitric oxide production by endothelium [7] and a long-term effect involving the mobilization of endothelial progenitor cells from the bone-marrow and subsequent regeneration of the endothelium [7,10]. In addition, it has been demonstrated that low doses of ESAs, including DarbEPO and carbamylated derivative (CEPO), are cytoprotective and may have utility in preventing ischemia-related progressive vascular injury and organ failure [44]. Specifically, an optimal dose of DarbEPO has been suggested for the treatment of myocardial ischemia/reperfusion models in mice (3.0 μg/Kg [45]) and rats (2.5 μg /Kg [46]). Different authors have reported that low-dose, but not high-dose, EPO administration improves myocardial infarction in patients with STEMI [47,48]. The potential adverse effects of EPO at high doses included vascular access thrombosis and other thrombotic complications. Furthermore, high doses of ESAs have been associated with elevated levels of inflammatory biomarkers that may contribute to the excess risk associated with high-dose ESA [49]. The selection of ESA doses that have protective effects with the understanding that “high is bad, low is good” may emerge from these clinical trials.

The balance between the benefits and risks in the use of epoetins most likely depends on several factors, including the effective drug dose, the duration of drug treatment, drug pharmacokinetics and the functional responsiveness of endothelial EPORs over time. These parameters could account for the differences in therapeutic effectiveness and side effects demonstrated for the different ESAs.

In this study, we demonstrate for the first time that three structurally different EPOR agonists, DarbEPO, EPOα and CERA, regulate the functional response of EPORs over time in different ways. In particular, we show that EPOα, DarbEPO and CERA, three clinically used ESAs, desensitize EPORs and display marked differences in EPOR resensitization kinetics, which is a critical process for restoring the functional response of the receptor at the membrane following prolonged agonist exposure and desensitization.

EPOR desensitization and resensitization were evaluated by assessing the intracellular STAT-5 phosphorylation/activation state. This phosphorylation pathway has been identified as one of the most important intracellular signaling pathways involved in EPOR-mediated effects in different cell types [21–24]. EPO-dependent tyrosine phosphorylation, nuclear translocation and activation of DNA binding of STAT-5 have been clearly demonstrated [50].

As a first step, we assessed whether and the mechanism by which EPOα, DarbEPO and CERA activate EPOR signaling in HUVECs. All agonists activated STAT-5 phosphorylation in a concentration-dependent manner with different kinetics. Both EPOα and DarbEPO showed a monophasic activation of STAT-5 phosphorylation peaking at a concentration of 1 IU/mL. CERA induced a more marked activation of STAT-5 with different, biphasic kinetics and a maximum at 5 IU/mL. These results suggest a different mechanism of action for DarbEPO and CERA at the receptor level. We hypothesize that CERA induces the recruitment of additional EPORs to the plasma membrane, thereby triggering the amplification of receptor signaling followed by a biphasic response.

To evaluate whether activation of the STAT-5 pathway induced by ESAs could have a functional role in endothelial cells, viability and angiogenesis assays were performed. We demonstrated that EPOα induced a significant increase in cell viability through a mechanism involving STAT-5 activation, which has yet to be demonstrated in other cell lines, such as trophoblasts [51] and endothelial cells [15]. Similar effects were obtained with DarbEPO, whereas CERA did not induce any significant change in cell viability. The inability of CERA to modulate cell viability after a long period of incubation with the HUVECs (72 h) could be caused by a distinct molecular mechanism of interaction of this derivative at the receptor level with respect to classical agents (see below) and the differential regulation of intracellular survival pathways.

Using the Matrigel *in vitro* angiogenesis assay model, we then investigated whether the different ESAs may directly affect vascular network formation. HUVEC incubation with EPOα, DarbEPO or CERA induced a significant increase in the cell number of mesh-like structures, a valid topological parameter of angiogenesis [52,53], suggesting that these compounds have pro-angiogenic effects on endothelial cells. However, because the STAT-5 inhibitor alone showed an inhibitory effect on endothelial cell remodeling (data not shown), it was not possible to demonstrate the involvement of this pathway in ESA-mediated angiogenic effects, although it has been reported that EPO is a pro-angiogenic factor in HUVECs and that the phosphorylation and nuclear translocation of STAT-5 is important for the effects of EPO on endothelial cells [15].

These data suggest that STAT-5 activation represents a good functional marker to investigate the regulatory mechanisms of EPOR in response to ESAs in endothelial cells.

Therefore, we investigated whether and the mechanism by which prolonged exposure of cells to EPOα, DarbEPO and CERA induces EPOR desensitization. For the first time, we demonstrated that incubation of HUVECs with these agonists for graded period of times induces significant receptor desensitization as evaluated by impairment of the receptor-mediated response. No significant differences in the desensitization kinetics induced by EPOα and DarbEPO were observed, suggesting that these two agonists act by similar mechanisms. By contrast, CERA caused a more rapid EPOR desensitization compared to the other ESAs. These differences could be explained by assuming that the different ESA derivatives act by distinct molecular mechanisms at the receptor level. Compared to EPOα, CERA has a lower affinity for its receptor and dissociates more quickly [54]. The lower receptor affinity could justify the need for a higher CERA concentration to obtain the maximal activation of intracellular signaling. Furthermore, the rapid association/dissociation kinetics of CERA could be the basis for the rapid EPOR-induced desensitization.

Finally, we assessed whether and the mechanism by which agonist removal after EPOR desensitization could restore receptor function. Among the regulatory mechanisms controlling the functionality of plasma membrane receptors, resensitization is a key event in ensuring the recovery of membrane receptors to an active state and allowing these receptors to respond to subsequent stimuli.

Different resensitization kinetics were observed after exposure to EPOα and DarbEPO for different times. When receptor desensitization was induced for a shorter period of time (18 min), receptor responsiveness recovery was observed after a 2 h wash-out period. By contrast, when receptor desensitization was induced for a longer period of time (54 min), receptor resensitization was detected only after a 24 h wash-out period. As expected from studies on other membrane receptors, these results demonstrate that the exposure-time and, to a lesser extent, the concentration of the agonist, are crucial factors that regulate the time needed for resensitization. When DarbEPO was used in the desensitization/resensitization experiments, the recovery of functional responsiveness occurred with significantly slower kinetics. At low DarbEPO doses and following short desensitization times, significant EPOR resensitization occurred only after a 6 h cell wash-out period. By increasing the DarbEPO concentration to 3 IU/mL and/or the desensitization period to 54 min, EPOR responsiveness appeared to be completely absent even after a 24 h wash-out period. When CERA was used in the desensitization experiments, the recovery of receptor responsiveness at the same desensitization times was faster for CERA compared to EPOα. Taken together, these results suggest that EPOα, DarbEPO and CERA differentially affect the intracellular machinery involved in the recycling of EPORs to the plasma membrane. It is likely that structural differences in the molecules may affect intracellular trafficking of the receptors and may change the permanence of the activated receptors at the cell surface. EPOR ubiquitination and internalization is a critical event controlling the amplitude and duration of EPO signaling [55], because the cell surface level of EPOR controls cellular EPO sensitivity [56] and endocytosis may lead to destruction of the activated protein complex to terminate signaling [57]. Furthermore, receptor internalization is a pre-requisite for receptor resensitization on the plasma membrane to return to a functionally active state. In this context, the different ESAs, which are characterized by strong, chemical structure variability, may affect the EPOR endocytic machinery and may differentially control receptor regulatory mechanisms. DarbEPO is biochemically distinct from EPO and has a higher molecular weight and a greater negative charge [38,58]. DarbEPO contains five *N*-linked carbohydrate chains, two more than EPO, and this chemical modification allows DarbEPO to carry a maximum of 22 sialic acid residues compared to EPO, which supports a maximum of 14 sialic acid residues. These molecular modifications to EPO determined a reduction in EPOR binding affinity for hyperglycosylated analogues and an increase in the ligand-receptor dissociation constant from the receptor with a consequent loss in the amount of ESA degradation. [59,60]. CERA was obtained by the introduction of a large methoxy-polyethyleneglycol polymer chain into the EPO molecule via amide bonds between the *N*-terminal group of alanine and the S-amino groups of lysine with a succinimidyl butanoic acid linker [61]. Receptor binding characteristics and the pharmacokinetics of CERA differ quite significantly from the first and second generation ESAs [62]. CERA has a lower affinity for its receptor and dissociates more quickly from the receptor [63]. It is hypothesized that the binding of CERA to its receptor is too brief to allow for internalization of the molecule, and this may account for the repeated binding, stimulation and dissociation that leads to prolonged activity *in vivo* and an extended elimination half-life. In addition, binding of CERA to its receptor could be too brief to allow for receptor internalization and may account for the rapid recovery of receptor functioning after desensitization.

To determine whether these ESAs may affect EPOR turnover as a consequence of different receptor binding mechanisms, we evaluated the effect of receptor desensitization/resensitization induced by the three epoetins on EPOR expression. We demonstrated that EPOR, after desensitization induced by EPOα, DarbEPO and CERA, is rapidly downregulated. After EPOα or CERA treatment, the receptor resensitized, and this process involved the synthesis of a new pool of receptor protein. Conversely, following treatment with DarbEPO, the physiological turnover of EPOR appeared to be impaired. In this case, there was no new EPOR synthesis, the levels of receptor protein remained low and the receptor was not able to recycle to the plasma membrane in an active functional state.

Based on these data, we hypothesize that the reversible desensitization of EPOR, other than serving to turn-off a biological response, may acts as an “on” switch to sustain long-term signaling and control of receptor expression at the nuclear level. Differences in the chemical structure of the epoetins most likely affects the endocytic machinery of EPOR and may differentially control EPOR turnover with important functional consequences for the receptor.

Further experiments investigating the intracellular route of EPOR following activation by the different ESAs are necessary to specifically identify the molecular mechanisms underlying the different effects of ESAs on EPOR regulatory mechanisms.

Taken together, our results demonstrate the following:

In HUVECs, the EPOR agonists EPOα, DarbEPO and CERA induced the time- and concentration-dependent stimulation of STAT-5 proteins.

ESAs modulate HUVEC viability through a mechanism likely involving STAT-5 phosphorylation, and ESAs also increase angiogenesis.

These agonists regulated the functional response of EPORs over time, causing time- and concentration-dependent receptor desensitization, with similar kinetics for EPOα and DarbEPO and more rapid kinetics for CERA.

The three epoetins displayed significant differences in receptor resensitization. The kinetics of receptor resensitization were strictly dependent on the type of agonist, agonist concentration and the duration of receptor exposure to the agonist. The rate of EPOR resensitization was greater for CERA compared to EPOα or DarbEPO and is slower when the duration of agonist exposure is prolonged. These differences could be ascribed to the differential regulation of EPOR turnover.

These data support the hypothesis that the activation of EPOR by low-dose ESAs for a short period of time is an essential pre-requisite for maintaining the receptor in a functional state in endothelial cells. These results may represent an important starting point for setting new therapeutic protocols for EPO and its derivatives in the treatment of vascular injury.

Although it may be difficult to translate our data into an *in vivo* model, the study of EPOR regulatory mechanisms in cells is a critical starting point for dissecting the functional responsiveness of EPORs during ESA activation for individual optimization of an efficacious therapy. Although the availability of long-lasting drugs is clinically useful in terms of cost and number of administrations, it should be noted that the choice of different ESAs and the use of high doses of these drugs may affect the physiological turnover of the receptor and may cause the onset of drug resistance with consequent drug dose increases to obtain similar pharmacological effects.

## 4. Materials and Methods

### 4.1. Materials

Recombinant erythropoietin (EPOα) was kindly supplied by Janssen-Cilag (Milan, Italy). Hyperglycosylated recombinant DarbEPO was purchased from Amgen Dompé SpA (Milan, Italy). CERA was purchased from Roche Diagnostic, Italy. Human umbilical vein endothelial cells (HUVECs) and cell culture medium (EGM^®^-2 BulletKit^®^) were purchased from Lonza srl (Milan, Italy). The RayBio^®^ Cell-Based STAT-5 (Tyr694) ELISA kit was purchased from RayBiotech Inc. (Norcross, GA, USA). The STAT-5 inhibitor *N′*-((4-Oxo-4H-chromen-3-yl)methylene)nicotinohydrazide was purchased from Calbiochem (Merck-Millipore, Nottingham, UK). All other chemicals were supplied by standard commercial sources.

### 4.2. Cell Lines and Drug Treatments

HUVECs were seeded onto 1% (wt./vol.) gelatin-coated tissue culture flasks and maintained in EGM^®^-2 BulletKit^®^ medium supplemented with 10% FBS. Cells (2500 cells/cm^2^) were grown at 37 °C in a humid atmosphere in the presence of 5% CO_2_. HUVECs were grown to 80% confluence and collected with the specific commercial solution, OneReagentPack™. The cells were used in successive experiments until the fourth detaching passage. The cells were treated with different ESA doses ranging from 1 IU/mL to 10 IU/mL. The doses of EPOα, DarbEPO and CERA used in the experiments were standardized based on the dose-conversion ratio (DCR) between EPOα and other ESAs as follows: 1 μg DarbEPO or CERA corresponds biophysically to 200 IU rhEPO peptide [64].

### 4.3. STAT-5 Phosphorylation Assay

To investigate EPOR-mediated STAT-5 phosphorylation, HUVECs were maintained in the appropriate serum and growth factor deprivation conditions (SFGD) prior to the assay. To determine the optimal experimental conditions for evaluating cell signaling caused by EPOR activation, the following three culture conditions were analyzed: (1) cells were starved for 12–24 h before the experiment using M199 medium devoid of FBS and growth factors and supplemented with 0.5% bovine serum albumin (BSA) and 0.2 mM sodium orthovanadate (OVD) (buffer 1); (2) cells were starved for 12 h before the experiment using M199 medium devoid of FBS and growth factors in the absence of BSA (buffer 2); and (3) cells were starved for 12 h before the experiment using M199 medium devoid of FBS and growth factors and supplemented with 0.2 mM OVD 30 min before experiments (buffer 3). For all experimental conditions, the effect of EPO (1 IU/mL) treatment for different periods of time (5–10 and 30 min) on STAT-5 phosphorylation was evaluated following the instructions provided with the kit. Briefly, following EPOR stimulation, HUVECs were fixed to preserve any protein modifications, including phosphorylation. After blocking, anti-phospho-STAT-5 (Tyr694) or anti-STAT-5 (primary antibody) was added to the wells. The wells were washed and HRP-conjugated anti-mouse IgG (secondary antibody) was added to the wells for one hour. After washing, TMB substrate solution was added to the wells and the color developed in proportion to the amount of protein. The stop solution changed the color from blue to yellow, and the intensity of the color was measured at 450 nm. The signals were normalized to the cell number in each well using a 0.1% crystal violet solution assay, and the data were then plotted.

In the experimental set, vascular endothelial growth factor (VEGF, 25 ng/mL) and granulocyte-macrophage colony-stimulating factor (GM-CSF, 5 ng/mL) were used as positive controls.

### 4.4. EPO-Mediated STAT-5 Phosphorylation: Concentration and Time Dependence

After determining the optimal experimental conditions, the concentration- and time-dependent activation of STAT-5 phosphorylation induced by the EPOR agonists, EPOα, DarbEPO and CERA, were evaluated. After serum starvation for 24 h in buffer 1, the cells were treated for different times (1–30 min) with EPOα (1 IU/mL, corresponding to 8.4 ng/mL), DarbEPO (1 IU/mL, corresponding to 5 ng/mL) or CERA (5 IU/mL, corresponding to 16.7 ng/mL) in buffer 1. In parallel, the same experiments were performed in the presence of the STAT-5 inhibitor *N′*-((4-Oxo-4H-chromen-3-yl)methylene)nicotinohydrazide (80 μM) [38]. The cells were then processed, and the levels of STAT-5 phosphorylation were quantified as described above.

### 4.5. Viability Assay

To evaluate the effects of the ESA derivatives on HUVEC viability, the cells were treated for 72 h with EPO (1 IU/mL), DarbEPO (1 IU/mL) or CERA (5 IU/mL) in the absence or presence of 80 μM STAT-5 inhibitor. Cell viability was determined by a tetrazolium salt-based assay (Promega CellTiter 96^®^ AQ_ueous_ Cell Proliferation Assay), according to the manufacturers’ instructions. The production of formazan in the cell culture was measured by absorbance at 490 nm using a microplate reader (Victor Wallac 2, Perkin Elmer, CA, USA).

### 4.6. *In Vitro* Angiogenesis Model

For the *in vitro* tube formation assay, HUVECs were seeded onto gelatin-coated 6-well plates and incubated with assay medium with the addition of drugs (EPOα 1 IU/mL, DarbEPO 1 IU/mL, CERA 5 IU/mL, with or without STAT-5 inhibitor). After 24 h, the cells were detached with trypsin-EDTA, and 7 × 10^4^ cells per well were seeded in 24-well plates coated with 250 μL 10 mg/mL Matrigel (BD Bioscence, Franklin Lakes, NJ, USA) that was thawed on ice at 4 °C and allowed to solidify at 37 °C for 30 min prior to cell seeding. After 20 h, the tube structures were observed with an inverted microscope (Hund, Wetzlar, Germany) equipped with a digital camera (Nikon, Sesto Fiorentino, Italy). Six fields (magnification 10×) were captured for each sample, performed in duplicate. For each image, the total length of the tube network and the number of intact loops were quantified with the image analysis software ImageJ (public domain, Image Processing and Analysis in Java, National Institutes of Health) using the plug-in AngioJ for the angiogenesis assay.

### 4.7. EPOR Desensitization

To measure desensitization, EPOR-mediated STAT-5 phosphorylation was evaluated following exposure of the cells to different EPOα, DarbEPO or CERA concentrations for different periods of time. Specifically, the concentration-dependence desensitization treatments were performed by incubating the cells with medium alone (control) or with different concentrations of EPOα, DarbEPO or CERA (0.5, 1, 2.5, 5, 10 I U/mL) for 60 min at 37 °C. For the time-dependence experiments, the cells were pre-incubated with medium alone (control) or with a selected agonist concentration (1 IU/mL for EPOα or DarbEPO and 5 IU/mL for CERA) for different periods of time ranging from 5 to 120 min. The cell culture medium contained sodium orthovanadate to avoid dephosphorylation of the receptor. Following desensitization, the cells were washed three times in buffer 1, and EPOR responsiveness was then assessed by evaluating the EPOα- (1 IU/mL for 5 min), DarbEPO- (1 IU/mL for 5 min) or CERA-induced (5 IU/mL for 5 min) phosphorylation of STAT-5.

### 4.8. EPOR Resensitization Experiments

HUVECs were treated for 18 or 54 min with buffer 1 alone (control), 1 IU/mL or 3 IU/mL EPOα or DarbEPO or 5 IU/mL CERA to induce receptor desensitization. Following desensitization treatment, the cells were washed and incubated with medium in the absence of EPOR agonists for 2 to 24 h for resensitization. Following this incubation, the recovery of receptor responsiveness was quantified by evaluating EPOα- (1 IU/mL for 5 min), DarbEPO- (3 IU/mL for 5 min) or CERA-induced (5 IU/mL for 5 min) phosphorylation of STAT-5. In the resensitization experiments, sodium orthovanadate was not included in the cell culture medium, because de-phosphorylation processes are required for the recovery of receptor responsiveness.

### 4.9. EPOR mRNA Expression

The relative mRNA quantification of EPOR was performed by real-time polymerase chain reaction (real-time PCR). HUVECs were treated for 54 min with complete medium (control), EPOα (1 IU/mL), DarbEPO (1 IU/mL) or CERA (5 IU/mL). Following treatment with the three epoetins for 54 min, aliquots of cells were incubated in medium alone for 24 h to allow for receptor resensitization. Total RNA was then isolated using the RNeasy^®^ Mini Kit. Total RNA (1 μg) was reverse transcribed using a QuantiTect Transcription Kit in a total volume of 20 μL. The RT-PCR mixture contained a total volume of 25 μL consisting of 12.5 μL DyNAmo Flash SYBR Green qPCR Mastermix (ThermoScientific), 5 μL cDNA, 3.5 μL H_2_O and 1 μM of the following primers: β-actin forward 5′-CGTCTTCCCCTCCATCG-3′ and reverse 5′-GCTTTTTTGTCCAGGCACTTCAT-3′ and EPOR forward 5′-AGCCCAGAGAGCGAGTTTGA-3′ and reverse 5′-CCACAGGCAGCCATCATTCT-3′. All reactions were performed for 40 cycles using the following temperature profiles: 98 °C for 30 s (initial denaturation), 60 °C for 30 s (annealing) and 72 °C for 30 s (extension). β-actin was used as the housekeeping gene. The mRNA levels for each sample were normalized against the β-actin mRNA levels, and the relative expression was calculated using the Ct value. PCR specificity was determined by both melting curve analysis and gel electrophoresis, and the data were analyzed by the standard curve method.

### 4.10. Statistics

Statistical analysis and graphical presentation were performed with Graph-Pad Prism 4 software (Graph-Pad Software Inc, San Diego, CA, USA, 2007). The data were analyzed using one-way analysis of variance (ANOVA) followed by Bonferroni’s multiple comparison test. A *p* value < 0.05 was considered statistically significant. All of the data are presented as the mean ± SEM.

## Abbreviations

EPO: erythropoietin
EPOR: erythropoietin receptor
DarbEPO: darbepoetin alpha
CERA: continuous erythropoietin receptor activator
ESAs: erythropoiesis-stimulating agents
HUVEC: human umbilical vein endothelial cells
